# Immune-Related Mendelian Randomization Signals for Anxiety Disorders Highlight the CD40 Locus on Chromosome 20 (chr20:46118–46130 Mb, GRCh38)

**DOI:** 10.3390/genes17091018

**Published:** 2026-08-27

**Authors:** Luwei Wang, Yifan Duan, Bo Sun, Sa-ouk Kang, Zheng Ye, Rui Liu

**Affiliations:** 1 School of Food Engineering, Yantai Engineering Research Center of Food Green Processing and Quality Control, Ludong University, Yantai 264025, China; xiaolingdangwang@gmail.com (L.W.);; 2 State Key Laboratory of Digital Medical Engineering, Southeast University, Nanjing 210096, China; sunbo@seu.edu.cn; 3 Laboratory of Biophysics, School of Biological Sciences, Institute of Microbiology, Seoul National University, Seoul 151-742, Republic of Korea; kangsaou@snu.ac.kr; 4 Institute of Computational Science and Technology, Guangzhou University, Guangzhou 510006, China

**Keywords:** anxiety disorders, Mendelian randomization, CD40, immune phenotypes, expression quantitative trait locus, colocalization, genetic epidemiology

## Abstract

Background/Objectives: Although genetic studies have linked immune phenotypes with anxiety, broad Mendelian randomization (MR) screens require explicit multiple-testing control. We reappraised 1753 reported immune-cell-trait-to-anxiety inverse-variance-weighted (IVW) association records available in the analysis tables and assessed the chromosome 20 signal that includes CD40. Methods: The reported records were derived from the 731-trait Sardinian flow-cytometry GWAS of Orrù et al. and were evaluated across one UK Biobank and three FinnGen anxiety phenotypes; outcome-specific Benjamini–Hochberg false-discovery-rate (FDR) correction was applied to the available analysis table. The leading locus was examined using bulk and single-cell expression quantitative trait locus annotations, two-sample MR, summary-data-based MR (SMR), HEIDI tests, and regional colocalization results. Results: Of 1753 tests, 120 were nominally significant, and 21 were retained after outcome-specific FDR correction (14 for UK Biobank anxiety and 7 for FinnGen all anxiety; none for generalized or phobic anxiety). B-cell and regulatory/CD4 T-cell traits were common among retained associations, and several B-cell traits mapped to variants at the CD40 locus. For FinnGen all anxiety disorders, monocyte instruments gave a positive estimate (beta = 0.00418, *p* = 9.25 × 10^−4^), whereas three naïve B-cell instruments gave a negative estimate (beta = −0.0520, *p* = 2.64 × 10^−4^). Monocyte SMR was null (*p* = 0.542). Naïve B-cell SMR was nominally significant (*p* = 0.00537), but HEIDI could not be calculated. Regional colocalization was weak in both cell contexts (PP.H4 = 0.0205 and 0.00272). Conclusions: The post-selection locus follow-up prioritizes a chromosome 20 immune locus but does not establish CD40-mediated regulation of anxiety. Because the upstream screen was not regenerated from source GWAS files and complete per-instrument F-statistics were unavailable, all findings are exploratory. CD40 remains a testable candidate for ancestry-matched fine-mapping and cell-state-specific validation.

## 1. Introduction

Anxiety disorders are common, clinically heterogeneous, and only partly explained by current biological models [1]. Immune changes have repeatedly been observed in people exposed to chronic stress or living with anxiety-related conditions, but these observations do not readily distinguish cause from consequence. Recent work instead points to coordinated communication among the brain, bone marrow, circulating immune cells, and lymphoid tissues [2,3]. This broader view has encouraged genetic studies of specific immune-cell traits rather than reliance on a few circulating inflammatory markers.

Mendelian randomization (MR) is often used to test whether genetically predicted variation in an exposure is associated with disease. Its appeal is clear, but large screens of hundreds of immune phenotypes create their own problems. The testing family must be defined before results are selected, genetic instruments must be handled consistently, and apparently supportive loci should be checked against pleiotropy and linkage disequilibrium (LD). These requirements are especially important in psychiatric genetics, where effects tend to be small and outcome definitions differ across cohorts [4,5,6,7]. Recent microbiota–anxiety and immune-cell–anxiety MR studies illustrate the breadth of phenotype screening and heterogeneity of anxiety definitions in this literature [8,9,10].

The present study reappraised the 1753 reported IVW records available in the supplied analysis table; it did not independently regenerate the upstream immune phenotype-outcome screen from source GWAS summary statistics. We applied Benjamini–Hochberg correction within each anxiety outcome and then asked which retained locus had enough supporting information to justify closer examination, and how far that information actually resolved the underlying gene.

Several B-cell traits pointed to chromosome 20 at the CD40 locus (GRCh38 chr20:46,118,240-46,130,378). We examined that region using molecular QTL, SMR/HEIDI, colocalization, and functional-annotation results. Our working expectation was that a convincing regulatory model would require agreement in effect direction, support from SMR with an interpretable HEIDI test, and evidence that the molecular and anxiety associations shared a causal variant. The results fell short of that standard, but they were informative about where the uncertainty lies.

## 2. Materials and Methods

### 2.1. Study Design and Inferential Scope

This was a structured reappraisal of a supplied summary-level IVW results table and a locus-prioritization study. We evaluated 1753 reported immune-to-anxiety IVW records spanning 731 immune phenotypes and four anxiety outcomes. Within each anxiety outcome, Benjamini–Hochberg FDR correction was applied to the available IVW records. The chromosome 20 analyses were evaluated as exploratory, post-selection gene prioritization rather than independent replication.

Although the cited source GWAS resources are public, complete SNP-level instrument files, harmonization logs, proxy decisions, and executable scripts required to regenerate every one of the 1753 records were not available in the materials used for this appraisal. We therefore did not independently re-clump, harmonize, or recalculate the full screen from source data.

### 2.2. Anxiety Phenotypes

Four anxiety phenotypes were evaluated. FinnGen release 12 included all anxiety disorders (finngen_R12_F5_ALLANXIOUS; 35,875 cases and 444,414 controls), generalized anxiety disorder (finngen_R12_F5_GAD; 7148 cases and 444,414 controls), and phobic anxiety disorders (finngen_R12_F5_PHOBANX; 6248 cases and 444,414 controls) [11,12]. These endpoints share controls and are partly nested, so they were compared as related phenotype definitions rather than independent replication samples. Individual-level case-overlap counts were not available in the public summary-statistics metadata; accordingly, we made no quantitative claim about the degree of case overlap. The UK Biobank/OpenGWAS phenotype ukb-d-KRA_PSY_ANXIETY was a European case–control summary association dataset with 1092 cases and 360,102 controls (total *n* = 361,194) [13]. The available OpenGWAS metadata identify the dataset as a binary Neale Lab analysis but do not fully specify the original ICD/self-report ascertainment; it was therefore interpreted only as a separately ascertained secondary outcome, not pooled with FinnGen or used to claim phenotype-specific replication.

### 2.3. Assembly of the Immune-to-Anxiety Result Set

The immune-to-anxiety master table contained 1753 inverse-variance-weighted (IVW) tests. Available fields included exposure and outcome identifiers, phenotype labels, numbers of variants, beta coefficients, standard errors, *p* values, confidence intervals, and immune-lineage annotations. Immune exposures were drawn from the 731 flow-cytometry traits in the Sardinian GWAS of Orrù et al. (approximately 3757 participants) [14]. The supplied table did not contain complete SNP-level exposure effect estimates and standard errors, instrument lists, clumping and harmonization logs, or per-instrument F-statistics for all 1753 records. We therefore could not independently verify instrument selection, LD clumping, allele harmonization, or instrument strength across the full screen. Outcome-specific Benjamini–Hochberg correction was applied to the available IVW records. Alternative MR estimators and standard sensitivity statistics were not available consistently and were not treated as confirmatory evidence.

The STROBE-MR reporting items and source-data Tables S8–S12 distinguish information available in the supplied materials from information that could not be independently reconstructed. They do not provide a complete per-instrument strength audit, harmonization log, or fully executable end-to-end pipeline for every reported association. Exposure-outcome sample overlap was not expected because the Sardinian immune GWAS and the FinnGen/UK Biobank outcome studies use distinct cohorts; however, this was not independently audited at the individual-record level.

### 2.4. Immune-Cell Classification and Cross-Method Summaries

Immune traits in the complete analysis table were grouped into B-cell, regulatory/CD4 T-cell, NK/NKT, other T-cell, and myeloid categories using the documented trait mapping. Counts by lineage were descriptive and were not formal enrichment analyses. Cross-method summaries were treated as a consistency layer only, not as independent validation.

### 2.5. Sparse-Instrument Colocalization Screen

Approximate Bayes factor colocalization was performed for selected trait-outcome pairs using the available MR instruments, following the framework of Giambartolomei et al. [15]. Because this sparse-instrument screen is not a dense regional analysis with ancestry-matched LD, it was used only for exploratory locus prioritization and not as evidence of a shared causal variant. Regional colocalization results were considered separately.

### 2.6. Molecular Annotation of the Chromosome 20 Locus

Bulk and single-cell expression quantitative trait locus (eQTL) annotations were obtained through xQTLbiolinks outputs and a local single-cell eQTL compendium [16]. ColocDB and downloaded SMR result collections were used as contextual resources. Because exact resource versions and preprocessing details were not available for all local downloads, these annotations were interpreted qualitatively rather than as independent replication. Because the immune exposure GWAS is Sardinian whereas the FinnGen and UK Biobank outcome cohorts are Finnish and British/European, respectively, ancestry differences and LD-dependent cross-cohort comparisons were treated as a limitation. Counts of supporting rows describe database coverage and should not be read as independent replication counts.

### 2.7. CD40 TSMR, SMR/HEIDI, and Regional Colocalization

For CD40, local monocyte and naïve B-cell exposure tables were evaluated with TSMR, manual SMR/HEIDI, and approximate Bayes factor colocalization. The available local tables indicated 50 monocyte and three naïve B-cell variants, but did not provide a complete per-variant strength audit. No LD matrix was available for the three naïve B-cell variants, and HEIDI could not be calculated for this analysis; all naïve B-cell estimates were therefore treated as sparse-instrument, exploratory results. The opposite directions observed in monocytes and naïve B cells were treated as unresolved rather than as evidence of cell-state specificity.

### 2.8. Interpretation of Evidence Layers

Evidence was interpreted hierarchically. Outcome-specific FDR-retained IVW results identified candidate associations but did not establish a common mechanism or independent replication across nested endpoints. TSMR provided cell-context-specific associations, SMR/HEIDI evaluated compatibility with a cis-regulatory model, and regional colocalization evaluated support for a shared causal variant. External QTL and sequence-prediction annotations informed biological plausibility only. No layer was counted as an independent replication when it reused the same locus, lead variants, outcome data, or overlapping molecular resources.

To examine directionality, the available reverse analysis used FinnGen all anxiety as the exposure and 695 immune traits as outcomes; no reverse IVW association was nominally significant, and none survived BH-FDR correction. Steiger filtering supported the recorded forward direction. A GWAS Catalog PheWAS [17] of rs1883832, rs4810485, and rs4239702 identified autoimmune and inflammatory associations (Supplementary Table S12); these results are treated as a pleiotropy warning rather than causal evidence.

## 3. Results

### 3.1. Outcome-Specific FDR Analysis Shows Restricted Immune Retention

**Figure 1:**
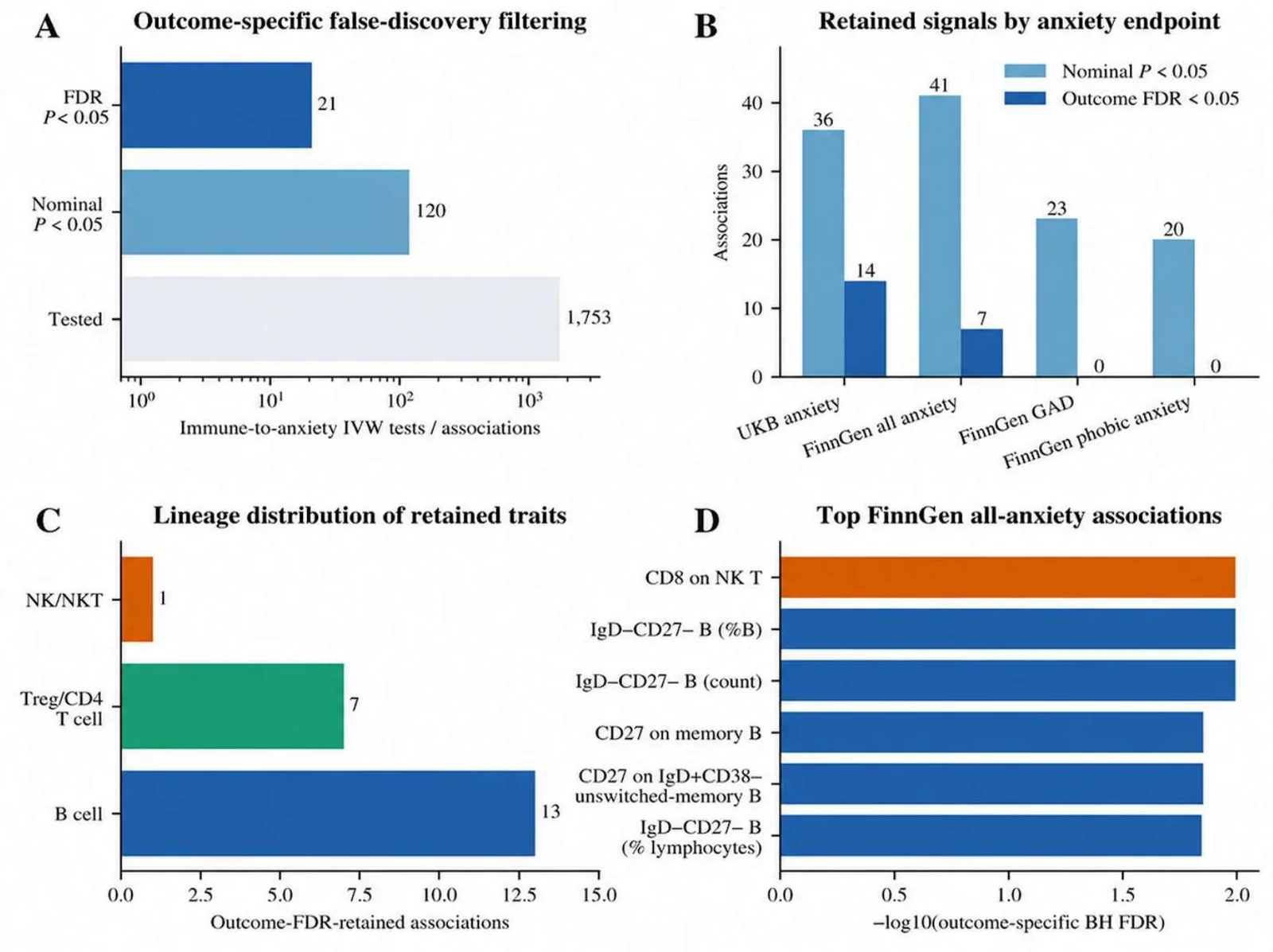
Outcome-specific FDR analysis identifies a restricted immune signal architecture. (**A**) Counts of tested, nominally significant, and outcome-specific FDR-retained immune-to-anxiety IVW associations across the available 1753-record analysis table. (**B**) Retained associations by anxiety endpoint. (**C**) Lineage distribution of the 21 retained associations. (**D**) Top retained immune-trait/outcome pairs. Pane (**A**) uses a log10 count scale. Microbiota analyses are not shown and do not contribute to this primary inference.

Across the 1753 IVW records available in the analysis table, 120 were nominally significant (*p* < 0.05). After Benjamini–Hochberg correction within each anxiety outcome, 21 associations were retained at FDR < 0.05: 14 for UK Biobank anxiety, seven for FinnGen all anxiety disorders, and none for generalized or phobic anxiety disorders. Figure 1 and Supplementary Table S1 show this outcome-specific retained set.

**Figure 2:**
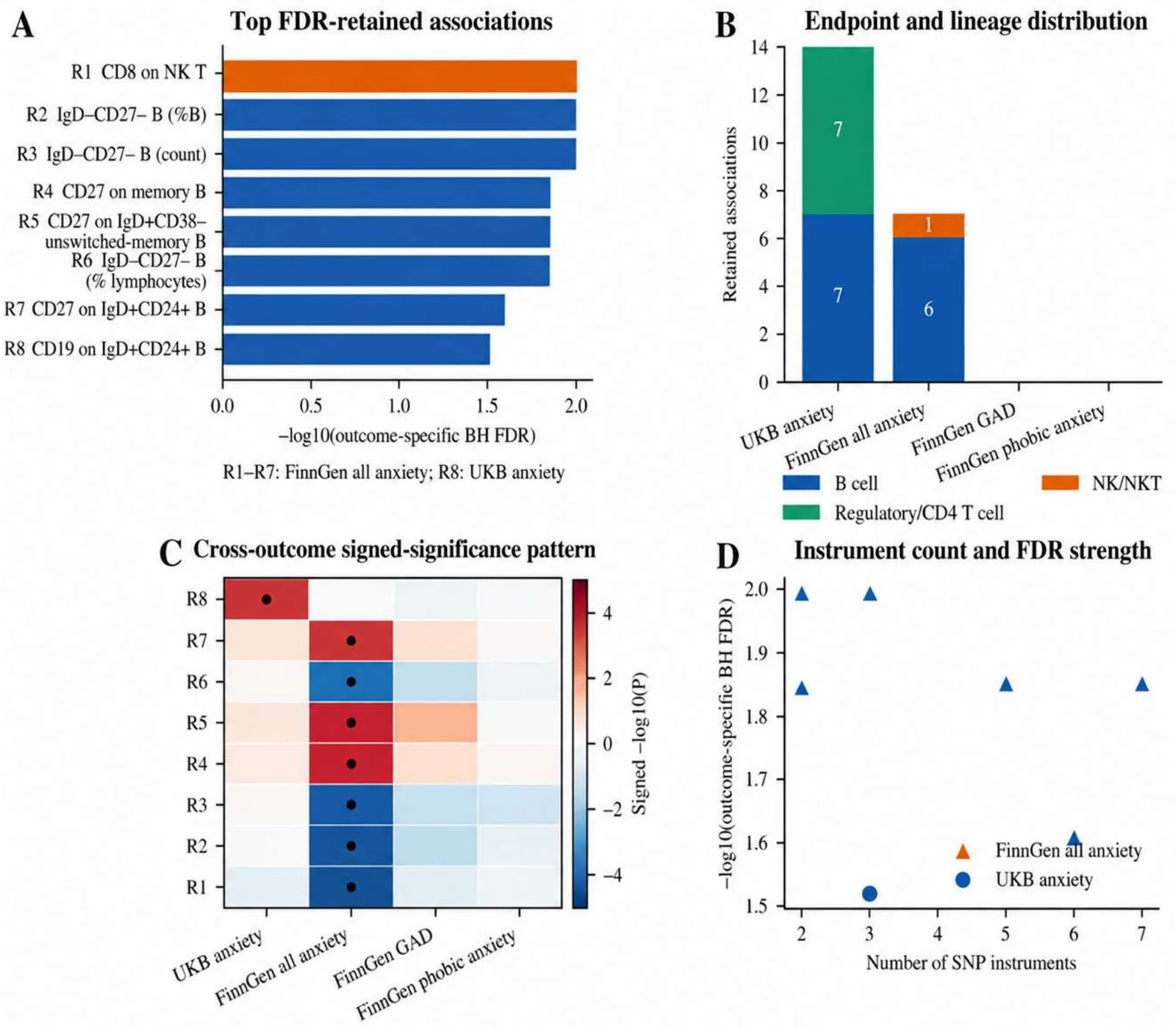
Outcome-specific retained immune landscape. (**A**) Top outcome-FDR-retained immune associations ranked by FDR strength and colored by lineage. (**B**) Endpoint and lineage distribution. (**C**) Signed cross-outcome pattern; red denotes positive effects, blue negative effects, and black dots denote outcome-FDR-retained associations. (**D**) Relation between instrument count and FDR strength. The panels are descriptive and do not establish independent replication across the nested FinnGen endpoints.

B-cell and regulatory/CD4 T-cell phenotypes appeared frequently among the retained associations (Figure 1). This pattern is descriptive rather than a formal lineage-enrichment test. The three FinnGen outcomes were not independent: they shared controls and represented related diagnostic definitions. UK Biobank provided a different ascertainment setting (Figure 2 and Supplementary Table S2).

### 3.2. The Leading Retained Signals Converge on a Chromosome 20 B-Cell-Related Locus

The highest-ranked retained rows included several measurements of CD27-positive, memory, or unswitched-memory B cells. Despite their different labels, these traits were indexed by variants in the same chromosome 20 region, particularly rs1883832 and rs4810485. The recurrence of one locus across correlated B-cell phenotypes motivated exploratory regional follow-up; because the same outcome data and significance-selected associations were reused, this did not constitute replication and may be affected by winner’s curse.

In the instrument-set screen, several B-cell trait-FinnGen all-anxiety pairs had PP.H4 values of approximately 0.84–0.85 (Supplementary Table S3). These estimates were based on the MR instruments alone and changed the priority of the locus, not the strength of the regional causal claim. Dense-region analyses were needed to ask whether the eQTL and anxiety associations were likely to share a causal variant.

### 3.3. CD40 Is a Leading but Unresolved Candidate Gene

CD40 was taken forward because immune-cell eQTL annotations linked variants in the region to its expression and because CD40 has well-established functions in B-cell activation and antigen-presenting cells [18,19]. The lead variant rs1883832 is the CD40 5′-UTR Kozak-sequence-1C>T variant with experimental evidence of altered CD40 translation efficiency [20]. Follow-up work also linked this Kozak polymorphism to tissue-specific CD40 expression in Graves’ disease [21]. The other lead variants, rs4810485 and rs4239702, are intronic CD40 variants; their GRCh38 coordinates, reference/alternative alleles, and functional annotations are provided in Supplementary Figure S7 and source data. The rs1883832/rs4810485/rs4239702 locus has prior autoimmune associations, including Graves’ disease, rheumatoid arthritis, multiple sclerosis, and inflammatory bowel disease [20,22,23]. No established CD40 polymorphism was identified as a specific anxiety-disorder variant in the prior literature. These observations make CD40 a sensible gene to test, but also make horizontal pleiotropy through autoimmune liability, disease consequences, or treatment a concrete alternative explanation. They do not exclude other genes in the interval.

Downloaded SMR collections contained CD40 entries from eQTL, methylation QTL, protein QTL, and splicing QTL datasets. A highlighted protein-QTL result at rs4810485 had *p*_SMR = 1.64 × 10^−4^ and an acceptable HEIDI result. CD40 had more annotated rows than SLC12A5, CDH22, SLC35C2, or NCOA5, but the underlying datasets were correlated and unevenly available across genes. We therefore report annotation breadth without treating row counts as statistical evidence.

Region-level xQTLbiolinks analyses did not yield strong colocalization for CD40 or the neighboring genes. AlphaGenome outputs were generated post hoc for selected variants in an immune-context subset; because the model version and subset specification were not fully documented, these predictions are reported only as supplementary hypothesis-generating annotation and are excluded from the primary gene-prioritization inference.

Table 1 summarizes the distinction between observations supported by the current analysis and claims that remain open. No deterministic gene score was used for inference because several components measure related features of the same locus.

### 3.4. Targeted Cell-Context Analyses Give Discordant Estimates

**Figure 3:**
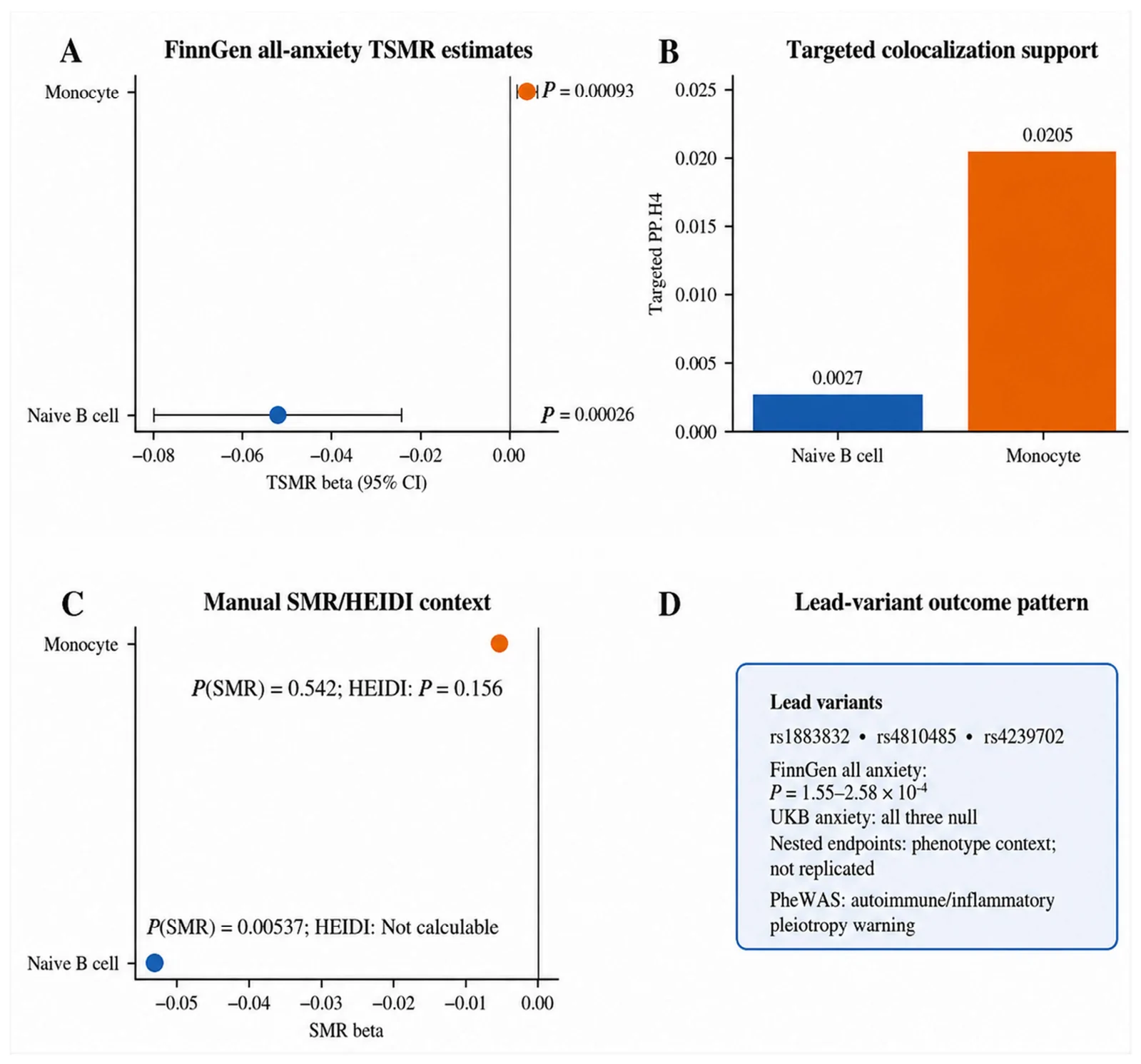
Discordant cell-context evidence at the chromosome 20/CD40 locus. (**A**) TSMR estimates for monocyte and Naive B-cell CD40 instruments for FinnGen all anxiety disorders differ in direction. (**B**) Targeted PP.H4 values are weak in both contexts. (**C**) Manual SMR results include a null monocyte estimate and a nominal Naive B-cell estimate without a calculable HEIDI test. (**D**) The lead-variant outcome pattern identifies association with broad FinnGen all anxiety, null UK Biobank results, and an autoimmune/inflammatory PheWAS pleiotropy warning.

For FinnGen all anxiety disorders, monocyte and Naive B-cell CD40 exposure tables were evaluated by TSMR, manual SMR/HEIDI, and targeted colocalization (Figure 3; Supplementary Tables S4 and S5).

For FinnGen all anxiety disorders, the monocyte analysis retained 50 variants and produced a positive TSMR estimate (beta = 0.00418, standard error = 0.00126, *p* = 9.25 × 10^−4^). The corresponding SMR estimate was close to zero and not significant (beta = −0.00547, *p* = 0.542; *p*_HEIDI = 0.156). Regional colocalization was also weak (PP.H4 = 0.0205).

The naïve B-cell analysis retained only three variants. Its TSMR estimate was negative (beta = −0.0520, standard error = 0.0142, *p* = 2.64 × 10^−4^), opposite to the monocyte estimate. SMR was nominally significant (beta = −0.0531, *p* = 0.00537), but HEIDI could not be calculated, and PP.H4 was 0.00272. The small instrument set and undocumented LD structure make this result particularly sensitive to individual variants.

Taken together, these analyses did not identify a consistent CD40 expression effect. Cell-state specificity is one possible explanation, but allele alignment, residual LD, horizontal pleiotropy, and selection of the locus remain viable alternatives. The available files cannot discriminate among them.

### 3.5. Cross-Phenotype Comparisons Do Not Provide Independent Replication

The monocyte estimate was positive for FinnGen generalized anxiety disorder (beta = 0.00410, *p* = 0.00111) but null for phobic anxiety and UK Biobank. Naïve B-cell estimates remained negative for the narrower FinnGen endpoints but were not nominally significant, and their PP.H4 values were approximately 1.4 × 10^−4^. These comparisons show how the result changes with phenotype definition; they are not independent replication tests.

The three lead variants—rs1883832, rs4810485, and rs4239702—were most strongly associated with the broad FinnGen endpoint (*p* = 1.55 × 10^−4^ to 2.58 × 10^−4^). Because the narrower FinnGen endpoints share controls and are largely nested within all anxiety, directionally similar estimates carry no additional replication information. All three variants were null in UK Biobank (Supplementary Table S6).

The chromosome 20 gene table is a descriptive annotation inventory (Table 2). Its priority tiers are labels, not statistical scores: Tier 1 denotes CD40 as the gene with the broadest convergent functional and molecular annotation, whereas Tier 2 retains neighboring genes as plausible alternatives. The categories are not probabilities of causality and do not provide independent confirmation.

**Figure 4:**
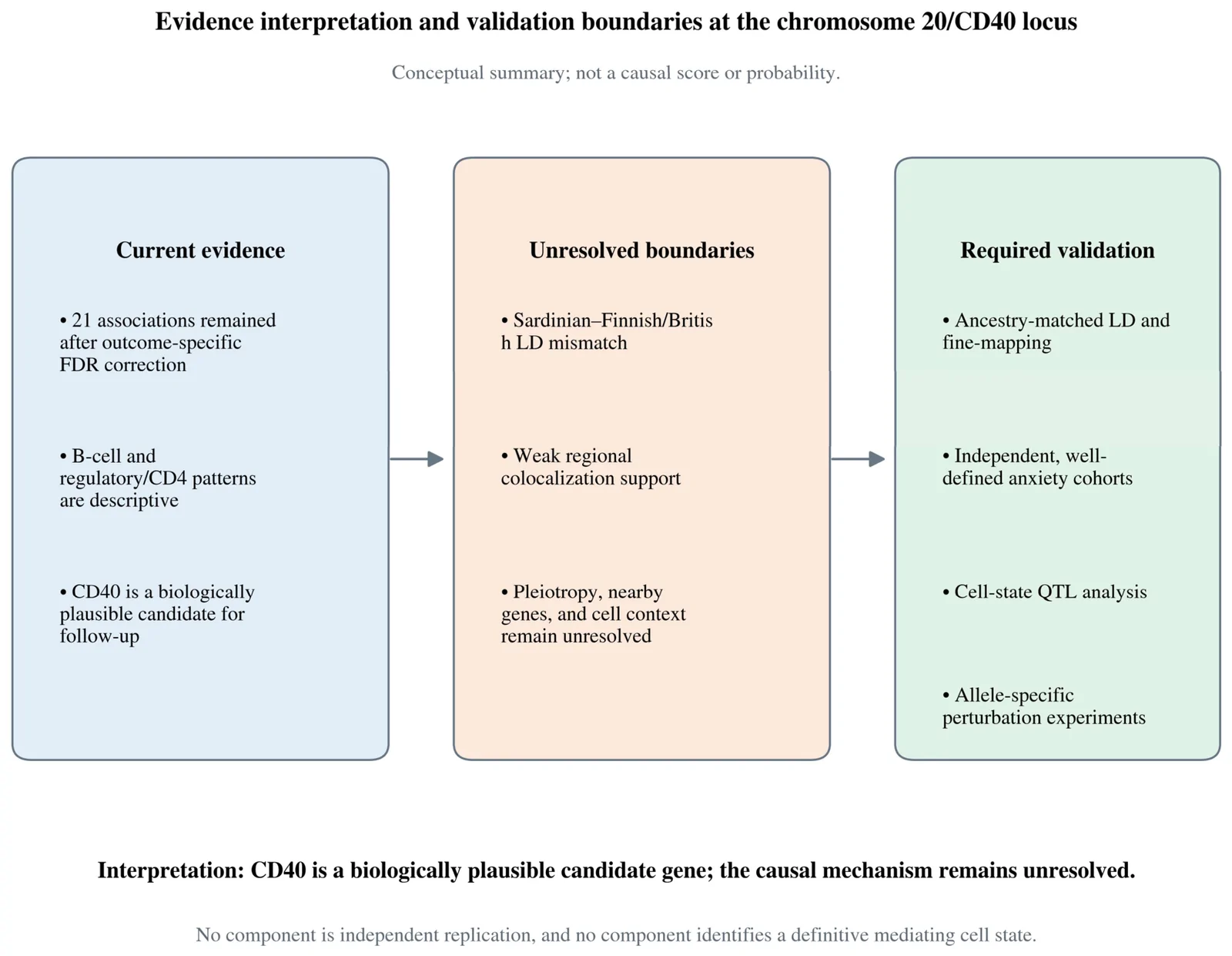
Interpretation boundaries for the chromosome 20/CD40 locus.

Figure 4 presents the interpretation boundaries for the chromosome 20 locus. It is a conceptual summary rather than a composite gene score, probability of causality, or independent confirmation of CD40.

The figure distinguishes the current evidence from unresolved LD, colocalization, pleiotropy, cell-state, and neighboring-gene questions, and identifies the validation steps required before a causal-gene claim can be made.

## 4. Discussion

The chromosome 20 signal is worth following, but the data do not support a definitive CD40-mediated interpretation. CD40 is an obvious candidate on biological grounds and is well represented in molecular QTL databases. Even so, the locus-level tests disagree in ways that matter: monocyte and naïve B-cell estimates point in opposite directions, monocyte SMR is null, the B-cell result rests on three variants without a documented LD matrix, and regional colocalization is weak.

The first limitation arises before the locus analysis. Outcome-specific FDR correction was applied to the available 1753-record analysis table, but the full upstream immune phenotype-outcome screen could not be regenerated from original GWAS files. Complete SNP-level instrument lists, harmonization records, and per-instrument F-statistics were not available for all records; this limits reproducibility and precludes independent verification of the full screen. The 1753 records should therefore not be interpreted as a newly regenerated screen or as evidence of a shared immune mechanism. For that reason, the apparent concentration of B-cell and regulatory T-cell traits should be treated as descriptive rather than as evidence of immune-lineage enrichment. Recent primers and reviews emphasize that the target estimand, instrument assumptions, analysis family, and reporting plan should be specified before results are selected [4,5,6,7].

The cell-specific analyses raise a different issue. A biological effect can certainly vary across immune-cell states, but opposite estimates are not, by themselves, proof of cell specificity. They can also result from differences in allele coding, LD, instrument selection, or horizontal pleiotropy. The extensive autoimmune literature at this locus makes autoimmune liability, disease consequences, and medication effects specific alternative pathways to test. Here, the null monocyte SMR result and the inability to run HEIDI for naïve B cells leave no firm basis for choosing one explanation over another. Potential sample overlap, directional pleiotropy, invalid instruments, outlying variants, and reverse direction should be examined with design checks and complementary estimators when the available instrument count permits [24,25,26,27,28].

Colocalization is the most direct challenge to a mediation claim. The PP.H4 values of 0.0205 in monocytes and 0.00272 in naïve B cells provide little support for a shared causal variant under the fitted model. The high values from the earlier instrument-set screen cannot offset this result because that screen used a small, selected subset of variants rather than dense regional data. SMR/HEIDI and regional colocalization address related but distinct questions, and neither can resolve a gene when the association is driven by different variants in LD or by multiple causal signals [29,30].

The external annotations do not settle the gene assignment. Genes with more QTL datasets and more detectable molecular phenotypes naturally accumulate more database entries. AlphaGenome is a post hoc computational hypothesis and is not part of the primary evidence hierarchy. SLC12A5 and CDH22 remain plausible regional alternatives, even though CD40 is a biologically compelling gene for targeted immune experiments. Large tissue atlases also show that regulatory effects depend on tissue composition and context, which complicates the interpretation of whole-blood support [31].

The cross-phenotype pattern also needs careful wording. The three FinnGen definitions share controls and may contain overlapping cases. Their similar lead-variant estimates are therefore internally consistent extensions of one cohort, not three replications. The UK Biobank result represents a separately ascertained case–control phenotype and should not be pooled with the FinnGen effects.

There is nevertheless a reasonable biological basis for further work. CD40–CD40L signaling regulates B-cell activation, antigen presentation, and communication between lymphoid and myeloid cells [18,19]. A useful next experiment would test the lead variants in stimulated and unstimulated B-cell and monocyte states, with allele-specific measurements of CD40 expression and downstream signaling. The most appropriate clinical follow-up would analyze well-defined anxiety subtypes in non-overlapping cohorts, rather than infer subtype specificity from the nested FinnGen endpoints. Recent single-cell studies have demonstrated that immune eQTL effects can be specific to cell type, activation state, or a continuous differentiation state, including transitions between naive and memory lymphocyte populations [32,33,34].

Nothing in the current analysis supports clinical testing or therapeutic manipulation of CD40 for anxiety. CD40 is a broadly acting immune regulator, and the direction of any anxiety-related effect remains uncertain. The practical outcome is a sharper experimental question, not a treatment recommendation.

A definitive end-to-end reanalysis would need to regenerate the source-data screen and report per-instrument F-statistics, the instrument threshold, clumping reference, proxy and palindromic-variant handling, sample sizes, and sample-overlap assessment in a completed STROBE-MR checklist. The present reappraisal cannot substitute for that procedure. Weighted-median and MR-Egger estimates, heterogeneity tests, tests of directional pleiotropy, Steiger filtering, reverse MR, and leave-one-out analyses should be provided whenever the number of instruments permits [4,24,25,26,27,28].

At the locus level, ancestry-matched fine-mapping and dense regional colocalization should be repeated with LD-aware, cell-state-specific QTL data. The monocyte-B-cell contrast should first be checked by allele-level harmonization and conditional analysis. Replication will also require anxiety cohorts that do not reuse the FinnGen controls or nested case definitions.

Study Limitations: The immune exposure GWAS was conducted in a Sardinian isolate, whereas the FinnGen and UK Biobank outcomes represent Finnish and British/European cohorts; this ancestry mismatch limits LD-dependent clumping, cross-cohort effect comparison, and colocalization. The FinnGen phenotypes were related rather than independent. At CD40, the naïve B-cell estimate used three variants without a documented LD matrix, HEIDI was unavailable, and the regional colocalization probabilities were low. External annotation counts may include correlated datasets. These constraints limit the locus analysis to exploratory prioritization. In addition, the 1753 reported IVW records were reappraised from the available analysis table rather than regenerated end-to-end from the original public GWAS files. The absence of complete SNP-level instrument data, harmonization records, and per-instrument F-statistics prevents an independent full-screen reproducibility and weak-instrument audit.

## 5. Conclusions

The outcome-specific FDR analysis identifies a restricted set of immune-to-anxiety associations and points to a chromosome 20 immune locus that merits further study. CD40 is a biologically credible candidate, but the present data do not establish it as the causal gene and do not support a CD40-mediated pathway for anxiety. Ancestry-matched LD-aware regional analyses, reverse-direction tests, and independent cell-state-specific validation are required before the claim can be strengthened. The absence of an executable SNP-level end-to-end pipeline and complete per-instrument F-statistics precludes independent verification of the full reported screen.

## Figures and Tables

**Table 1:** Evidence-to-claim matrix for the exploratory interpretation.

Evidence Layer	Main Result	Supports	Does Not Establish
Global MR analysis	A restricted set of immune-to-anxiety associations remained after outcome-specific FDR correction.	A transparent screening framework and candidate associations for follow-up.	A broad immune mechanism, formal lineage enrichment, or independent replication across anxiety outcomes.
Immune architecture	FDR-retained associations were descriptively concentrated in B-cell and regulatory/CD4 T-cell phenotypes.	Descriptive prioritization of phenotype classes.	Formal enrichment, a uniform immune signature, or causal relevance of any individual cell phenotype.
Sparse-instrument coloc screen	Several leading B-cell traits showed high PP.H4 in a sparse instrument-set screen.	Prioritization of a chromosome 20 lead cluster for regional follow-up.	Dense-region shared-causal-variant evidence or CD40 mediation.
Local sceQTL + TSMR/SMR	Naive B-cell analyses showed a nominal SMR signal but used three instruments and lacked HEIDI; monocyte analyses provided broader instrument coverage but null SMR evidence.	Cell-context hypotheses for follow-up.	A definitive mediating cell state, directionally consistent effect, or causal regulatory pathway.
External QTL and functional annotations	CD40 had broad molecular QTL and functional annotation, while neighboring genes retained support.	CD40 as a biologically plausible candidate gene.	Exclusion of neighboring genes, independent replication, or a resolved causal mechanism.

**Table 2:** Descriptive molecular-annotation inventory for chromosome 20 candidate genes.

Gene	Tier	Use	SMR Status	Rows *	Sequence-Model Note	Best Modality	Best *p*
CD40	Tier 1	Candidate only	Contextual	47	Hypothesis only	pQTL	1.64 × 10^−4^
SLC12A5	Tier 2	Neighbor retained	Contextual	7	Background	eQTL	4.76 × 10^−5^
CDH22	Tier 2	Neighbor retained	Contextual	6	Background	eQTL	6.47 × 10^−3^
SLC35C2	Tier 2	Neighbor retained	Contextual	4	Not assessed	eQTL	2.58 × 10^−2^
NCOA5	Tier 2	Neighbor retained	Limited	1	Weak	mQTL	4.04 × 10^−5^

* TSMR, two-sample Mendelian randomization; SMR, summary-data-based Mendelian randomization. Priority tier is descriptive only: Tier 1 indicates the leading candidate for follow-up; Tier 2 indicates retained neighboring candidates. SMR-row counts and sequence-model annotations reflect unequal database coverage and are neither causal probabilities nor independent replication.

## Data Availability

The available analysis tables and supporting code fragments can be obtained from the corresponding author upon reasonable request; they do not constitute an executable end-to-end pipeline for regenerating all reported associations. The immune-cell GWAS summary statistics are available from Orrù et al.; anxiety GWAS summary statistics are available from FinnGen release 12 and OpenGWAS/UK Biobank; molecular QTL summary statistics are available from their original public repositories, subject to their access terms.

## Supplementary Materials

The following supporting information can be downloaded at https://www.mdpi.com/article/10.3390/genes17091018/s1.

## Abbreviations

The following abbreviations are used in this manuscript:

BH	Benjamini–Hochberg
eQTL	expression quantitative trait locus
FDR	false-discovery rate
GAD	generalized anxiety disorder
GWAS	genome-wide association study
IVW	inverse-variance weighted
LD	linkage disequilibrium
MR	Mendelian randomization
PheWAS	phenome-wide association study
SMR	summary-data-based Mendelian randomization
TSMR	two-sample Mendelian randomization
