# Supplementary material for: Immune-Related Mendelian Randomization Signals for Anxiety Disorders Highlight the CD40 Locus on Chromosome 20 (chr20:46118–46130 Mb, GRCh38)
Source: Genes (Basel). 2026 Aug 27;17(9):1018. doi: 10.3390/genes17091018 (PMC13606397; doi:10.3390/genes17091018)
Supplement: Supplementary file 1 [file genes-17-01018-s001.zip › Supplementary_Checklist_S13_STROBE_MR.pdf]

Supplementary Checklist S13

STROBE-MR Checklist

Purpose. This checklist maps the main reporting items for the exploratory two-sample Mendelian-randomization (MR) analyses to the revised manuscript and supporting source tables. It records limitations transparently rather than inferring unavailable metadata.

No.	STROBE-MR item	Location	Status / information reported
1	**Title and abstract**	Title; Abstract	Reported. The title identifies genetic signals and locus prioritization; the abstract describes the analysis as exploratory and does not assert causality.
2	**Scientific background and rationale**	Introduction, paragraphs 1–4	Reported. Immune-cell traits, anxiety outcomes, CD40 biology, and the assumptions/limits of MR are introduced.
3	**Objectives and estimands**	Introduction, final paragraph; Methods 2.1	Reported. The global screen and locus follow-up are framed as exploratory prioritization, not confirmatory causal estimation.
4	**Study design and setting**	Methods 2.1–2.3; Table S10	Reported. FinnGen R12 endpoints and the separate UK Biobank/Neale Lab outcome are named. The source-table audit accompanies the manuscript.
5	**Participants and data sources**	Methods 2.2–2.3; Tables S9–S11	Reported with limitations. The exposure source is Orrù et al. (Sardinian cohort, n=3,757); Finnish and UK Biobank outcome contexts are stated. The exact UK Biobank ICD/self-report algorithm and individual-level FinnGen case overlap were not available in the retained metadata and are not inferred.
6	**Instrument selection**	Methods 2.4; Table S9	Reported with a remaining granularity limitation. SNPs were selected at $P<5\times10^{-8}$ and clumped at $r^2<0.1$ within 500 kb using a 1000 Genomes European reference. Table S9 reports instrument-set F summaries (minimum F=29.855; no audited set had F<10); a single-SNP F table is not separately supplied.
7	**Variant harmonization**	Methods 2.4; Table S9	Reported. Alleles were harmonized, palindromic variants were assessed using allele frequency where available, and unresolved ambiguous variants were excluded.
8	**Proxy variants and missing data**	Methods 2.4; Table S9	Reported as an audit limitation. The retained primary set is presented as directly harmonized variants; no proxy-substitution result is used for the main interpretation. Any unavailable/ambiguous variants were excluded rather than imputed.
9	**Sample overlap**	Methods 2.5; Discussion, limitations	Reported with limitation. Exposure and outcome cohorts are distinct by design (Sardinian, Finnish, British), so direct participant overlap is not expected; this does not remove ancestry/LD mismatch.
10	**Primary statistical methods**	Methods 2.4–2.6; Figure 1; Table S8	Reported. IVW was the primary estimator. Benjamini–Hochberg correction was applied within outcome-specific test families and across all 1,753 tests; 21 and 3 associations, respectively, met FDR<0.05.
11	**Sensitivity analyses and pleiotropy**	Methods 2.5–2.7; Results 3.4; Discussion	Reported with limitations. SMR/HEIDI, targeted colocalization, and external QTL annotation are reported where available. Sparse instruments and unavailable LD information precluded uniform sensitivity analysis.
12	**Directionality and reverse causation**	Methods 2.6; Discussion, limitations; Table S12	Reported as exploratory. Reverse-direction and Steiger checks are described, while reverse causation remains a live alternative because anxiety, stress, and medication may affect immune-cell composition.
13	**Colocalization and LD assumptions**	Methods 2.6; Results 3.3–3.4; Discussion	Reported with limitation. Colocalization is targeted and sparse-instrument based; cross-ancestry LD mismatch prevents interpreting it as definitive shared-causal-variant evidence.
14	**Phenotype dependence / non-independence**	Methods 2.3; Results 3.2; Discussion	Reported. FinnGen all-anxiety, GAD, and phobic-anxiety endpoints share a cohort and overlapping controls; narrower outcomes are not treated as independent replication. Exact individual case overlap is unavailable.
15	**Selection and multiplicity**	Methods 2.6; Results 3.3; Discussion	Reported. The chr20/CD40 follow-up reuses significance-selected signals and outcome data. Effect rankings and within-subset FDR values may be winner's-curse inflated and anti-conservative.
16	**Results**	Results 3.1–3.4; Figures 1–3; Tables S8–S12	Reported. The complete screen comprises 1,753 tests, with 120 nominal, 21 outcome-FDR-retained, and 3 global-BH-retained associations. Discordant monocyte/naïve-B-cell results are retained and interpreted cautiously.

No.	STROBE-MR item	Location	Status / information reported
**17**	**Data availability and reproducibility**	Methods 2.1; Data Availability Statement; Supplementary source tables	Reported. Public GWAS/QTL sources are identified. The revised analysis does not claim regeneration of every upstream source analysis; unavailable metadata are explicitly separated from results.
**18**	**Interpretation and generalisability**	Discussion; Conclusion; Figure 4; Table 1	Reported. CD40 is prioritized as a plausible candidate, not established as a causal mediator. Ancestry-matched fine-mapping, independent cohorts, cell-state QTL data, and perturbation experiments are required.

Abbreviations: FDR, false-discovery rate; HEIDI, heterogeneity in dependent instruments; IVW, inverse-variance weighted; LD, linkage disequilibrium; MR, Mendelian randomization; QTL, quantitative trait locus; SMR, summary-data-based Mendelian randomization.
